# Association between Lifestyle and Hypertension in Patients Referred to Health Care Centers of Ilam City in 2014

**DOI:** 10.5539/gjhs.v8n6p161

**Published:** 2015-10-26

**Authors:** Zahra Shafieyan, Mostafa Qorbani, Babak Rastegari Mehr, Mohammad Mahboubi, Aziz Rezapour, Omid Safari, Hossein Ansari, Maryam Esmaeli Kia, Hamid Asayesh, Morteza Mansourian

**Affiliations:** 1 Student Research Committee, Ilam University of Medical Sciences, Ilam, Iran; 2 Departments of Community Medicine, School of Medicine, Alborz University of Medical Sciences, Karaj, Iran; 3 Non-Communicable Diseases Research Center, Endocrinology and Metabolism Research Institute, Tehran University of Medical Sciences, Tehran, Iran; 4 Abadan School of Medical Sciences, Abadan, Iran; 5 Department of Health Economics, School of Health Management and Information Sciences, Iran University of Medical Sciences, Tehran, Iran; 6 Health Management and Economics Research Center, Iran University of Medical Sciences, Tehran, Iran; 7 Departments of Pediatrics, School of Medicine, Alborz University of Medical Sciences, Karaj, Iran; 8 Health Promotion Research Center, Zahedan University of Medical Sciences, Zahedan, Iran; 9 Department of Medical Emergencies, Qom University of Medical Sciences, Qom, Iran; 10 Public Health Department, Ilam University of Medical Sciences, Ilam, Iran

**Keywords:** lifestyle, hypertension, physical activity, stress, health responsibility

## Abstract

**Introduction:**

Lifestyle is referred to an individual’s healthy and unhealthy behaviors that can affect their health statues. The present study aim was association between lifestyle and hypertension in patients referred to healthcare centers of Ilam city in 2014.

**Materials and Methods:**

This research study was a case-control study. The data were collected through a standard questionnaire of health-promoting lifestyle profile (HPLPII) as well as the researcher’s direct visit to the health care centers in the city of Ilam. After the questionnaires were collected and classified, the data were entered into SPSS software and analyzed by descriptive statistics, chi-square tests, T-Tests and logistic regression.

**Results:**

The mean and the standard deviation of the age of the main and the control groups were 57.1 (2.22) and 56.5 (2.99) years old, respectively. 10.9%of the control group and 25.5. % of the cases was smoking cigarettes or hookah. The results of the data analysis showed that the mean scores obtained by the main and the control groups on measures of physical activity, psychological growth, stress and total lifestyleare significantly different, so that the obtained score in the dimensions in patients with hypertension was significantly lower than the score obtained among the healthy individuals.

**Conclusions:**

According to the results it seems that educational interventions in the field of healthy lifestyle for individuals with hypertension risk can have an effect on controlling this disease and reducing its incidence.

## 1. Introduction

Lifestyle is referred to an individual’s healthy and unhealthy behaviors that can have an impact on their health statues. A health-promoting lifestyle is one in which self-initiated, continuous, daily activity is undertaken with the deliberate aim of increasing or promoting an individual’s health and well-being (Hua et al., 2015). Studies have revealed that the most important health risk factors include poor diet, inactivity and low physical activity, as well as smoking which are the main factors for an individual’s lifestyle. Studies have also revealed that 80% of heart diseases and 90% of type-II diabetes could be avoided by making changes in these risk factors. Mean while, 1.3% of cancer cases can be prevented by improving nutrition and controlling weight; and 3.1% of other cases are preventable by avoiding smoking and changing lifestyles (WHO, 2002; Borhani et al., 2015).

Controlling risk factors in lifestyle and health habits such as poor nutrition, lack of exercise, as well as smoking, alcohol and drug use can almost decrease nearly 50% of premature death (Pescatello et al., 2015).

The statistics on causes of mortality indicate that 53%of causes of death are related to lifestyle, 21% to environmental factors, 16% to genetic factors and 10%to healthcare service systems (Long et al., 2000). Adopting each kind of lifestyle by an individual may predispose them to a range of diseases including hypertension which has been proposed as a major health problem and as a silent killer which is asymptomatic. If untreated, it will have lethal effects. Among the complications of hypertension; cardiovascular diseases including coronary heart disease, minimum heart failure, peripheral vascular diseases and chronic renal failure have been reported (Long et al., 2000; Mohamadi Zaid et al., 2006).

High blood pressure is the biggest international only-factor of diseases and death tolls. These non-communicable disease conditions are a major public health challenge and lead to heavy burden on society (Najar et al., 2005). It is predicted that the number of people with hypertension will increase in the next decade. In the United States, about 77.9 million (1 out of every 3) adults have hypertension (Dizaji et al., 2014; Hu et al., 2015). Therefore, there is a need to implement preventive strategies particularly in developing countries.

The prevalence of hypertension across the world is different. In rural regions in India, it is reported as 3.4% in men and 6.8% in women. The highest incidence in the world is reported in Poland, which is 46%. In the U.S., 58 million Americans are inflicted with hypertension disease (Poulter et al., 2015). In Iran, the prevalence of hypertension has been reported in different studies. This is 20.88% in Gonabad (Kearney et al., 2004), 14% in Kermanshah (Naghavi et al., 2001), 32% in Tehran (Azizi et al., 2008), 20% in Tabriz (Fatahi et al., 2000), 11% in Isfahan (Fakhrzadeh et al., 2002), and 15% in Yazd (Morvati et al., 2005) which imposes high financial burden on the country to treat and take care of patients with this disease. Given the importance of controlling the hypertension disease, the aim of this study was association between lifestyle and hypertension in patients referred to healthcare centers in Ilam city in 2014 to employ the findings in order to improve planning for lifestyle improvement and provide better services to patients.

## 2. Materials and Methods

This research study was a case-control study. The data in this study were collected through questionnaires and by the researcher’s direct visit to healthcare centers in the city of Ilam. The questionnaire is an international standard questionnaire on lifestyle entitled Health Promoting Lifestyle Profile (HPLP II) whose reliability was confirmed via Cronbach alpha 0.78 in the study by Morovati (Morvati et al., 2005) and its validity was approved by health education specialists. The questionnaire consists of two parts including demographic questions and health-promoting behavior questionnaire with 52 items with 4 responses, i.e. never-sometimes-often-always. The health-promoting behavior questionnaire measures the frequency of health-promoting behaviors in 6 dimensions including health responsibility (HR) (9 items), physical activity (PA) (8 items), nutrition (N) (9 items), spirituality (S) (9 items), interpersonal relations (IR) (9 items) and stress management (SM) (8 items). The range of health promoting lifestyle profile questionnaire score was between 52 to 208 and a separate score is calculated for each dimension.

The method of data collection involved the researcher’s visit to the healthcare centers under study, then the hypertensive patients were identified and the data were collected on the basis of data proportional allocation method. In this method, a proportion of the general population of the sample was allotted to each center. The patients’ blood pressure was recorded by health professionals’ reference to healthcare centers and the questionnaires were completed.

The same number of control group was selected among those referring to the centers and they completed the questionnaires, too. A matching was conducted in the case of control group that is the controls were selected in accordance with the main group as far as possible in terms of age, sex and place of residence and they only had one difference, i.e. the absence of hypertention disease. It should be noted that, since a number of participants were illiterate, the data were collected in the form of interviews.

According to the type of study and by taking the significance level of 5% and 90% test power as well as the use of appropriate statistical formula into account, the sample size in this study was 200 for every group and 400 in total.

Consulting with a statistician to analyze the data, firstly the total scores and then the scores for the mentioned six measures were calculated. After collecting and sorting the questionnaires, data were fed into the SPSS software and were analyzed by descriptive statistics as well as chi-square test, t-tests and logistic regression.

## 3. Results

The mean and the standard deviation (SD) age of the cases and the controls were 57.1 (2.22) and 56.5 (2.99) years old, respectively. The minimum and the maximum age of the participants in the control group was 29 and 79 years old and in case group was 29 and 91 years old, respectively.

41.1% of the control group and 38.9% of the cases were male and the rest were female. 77.6% of the control group and 80.3% of the patients were married. The number of family numbers for 63% of the control group was 4 or lower; it was 53.6% for the case group. The mean weight for the control group was 68.75 (SD 1.42) and for the case group, it was 71.58 kilograms (SD 1.62). The mean heights for the control group and the case group were 1.62 and 9.9 meters and 1.59 and 8.6 meters, respectively. The body mass index (BMI) in the control group was 32.3% underweight, 53.6% with a normal BMI and 14.1% were overweight or obese, while in the case group, these figures were 20.7%, 51% and 27.9%; respectively.

In patients with hypertension, 34.6% of them were diagnosed with hypertension and 65.4% had been diagnosed with symptoms. Headache with 77.4%, vertigo 21.2% and visual impairment with 1.4% were the most important symptoms of hypertension among the patients under study.

Regarding smoking, 10.9% of the control group and 25.5% of the main group were smoking cigarettes or hookah. 65.1% of the control group and 81.7% of the control group did not exercise during the week and the rest did regular exercises. 47.4% of the control group had less-than-eight-hour sleep per day and 52/6% slept more than eight hours while in the case group, 63.5% slept less than eight hours and 36.5% slept more than eight hours a day. In terms of the rate of salt intake, in the control group; 17.2% tended to use low-salt foods, 74% used foods with moderate salinity, and 8.8% had a tendency to have salty foods; while in the case group, the tendency to have low-salt foods, foods with moderate salt, and salty foods were 24%, 72.1% and 3.9% respectively. In terms of oil use in food; in the control group, 15.1% tended to have low-fat foods, 53.6% moderate foods, 30.12% had a tendency to use fatty foods. These figures in the main group were 27.9%, 41.8%, and 30.2%, respectively. In terms of temperament in the control group, 46.4% knew themselves as quiet individuals, 44.3% as kind of angry and 9.4% as angry. These figures for the case group were respectively 39.9%, 44.7% and 30.2%.

The results of the data analysis showed that the mean scores obtained by the case and control groups in PA, S, SM and lifestyle dimensions were significantly different, so that the total score obtained in the mentioned scales among patients with hypertension was significantly lower than the control group. The results are illustrated in Table 1.

**Table 1:** Compression of total lifestyle and the dimensions mean score in case and control group

	Case group		Control group		P-value

	Mean	SD	mean	SD	
TL	124.04	18.65	128.8	18.50	0.013
HR	16.8	4.27	16.89	4.66	0.84
PA	11.05	7.79	13.47	5.29	0.000
N	26.35	3.47	26.65	3.74	0.78
S	25.53	4.52	25.58	5.0	0.030
IR	22.55	4.19	22.80	4.20	0.56
SM	19.07	4.80	21.59	4.69	0.000

In addition, the t-test results revealed that the mean score of TL and its six dimensions among smokers was significantly lower than that of non-smokers. The mean score of the TL and the dimensions of PA, SM, N, IR, and health responsibility were significantly lower than smokers. The results are presented in Table 2.

**Table 2:** Compression of total lifestyle and the dimensions mean score in smokers and non smokers

	Smokers		Non smokers		P-value
	
	mean	SD	mean	SD	
TL	112.04	16.59	129.5	17.7	0.000
HR	14.29	4.14	17.43	4.33	0.000
PA	10.31	3.12	12.65	5.44	0.000
N	24.90	3.06	26.74	3.63	0.21
S	23.25	4.20	25.66	4.71	0.000
IR	19.86	3.89	23.31	4.33	0.000
SM	17.02	4.40	20.02	4.72	0.000

The results of the logistic regression showed that the relationship between lifestyle total score and lifestyle in the dimensions of PA, SM and S was statistically significant with hypertension (p<0.05). That is, as one score increases to the HPLP questionnaire, the risk of hypertension in the TL dimension (0.986), in the dimensions of PA (0.908), S (0.954) and SM (0.986) is reduced. These results are summarized in Table 3.

**Table 3:** The relationship between total lifestyle and the dimensions with chance of hypertension in logistic regression

	Case group	Control group	P-value	Odd ratio	Confident interval
TL	124.6	127.8	0.013	1.986	0.976- 0.997
PA	11.05	13.47	0.000	1.108	0.870- 0.947
S	25.53	26.58	0.032	1.21	0.914- 0.936
SM	19.07	21.59	0.000	1.486	0.858- 0.936

## 4. Discussion

Having health-promoting behaviors is one of the best ways by which people can control their health. Lifestyle should be considered as a complex combination of tasks and behavioral habits among individuals and groups particularly with regard to cultural infrastructure and socioeconomics conditions as well as social relationships and their personality.

In this study, 25.5% of the case group and 10.9% of the control group were taking cigarettes or hookah. These findings are not in line with the results provided by Mansourian (Mansourian et al., 2012) in the city of Gorgan in which 12% of patients and 20.5% of the control group smoked hookah and cigarettes. The results of the present study also do not confirm the findings in a study by Moghimi et al. (2007) among the elderly in the city of Yasouj where in 32.2% of the cases and 37.7% of the control group were cigarette smokers. This difference seems to be associated with the research environment in the aforementioned studies and the current study.

In the present study, 34.9% of controls and 19.3% of the cases did regular exercises during the week; these findings are somewhat in agreement with the results revealed by Najjar (Najar et al., 2005), in which the relationship between lifestyle and initial hypertension in the city of Sabzevarwas determined, because 42% of the control group and the 34.2% of the experimental group exercised during the week. The results are also somewhat similar to the findings in the study by Mansourian. (Mansourian et al., 2012) in which 39.5% of the control group and 17.9% of the cases did exercises during the week.

In terms of using foods high in salt, 24% of the cases and 17.2% of the control group had a tendency to have salty foods. This result which was in line with Najjar et al. (2005) study was 45% and 17.4%, respectively.

In the present study, 14.1% of the control group and 27.9% of the cases were obese. In the study conducted by Mansourian (Mansourian et al., 2012); 63% of the control group and 59% of the case group were obese which is much higher than the results of the present study. As well, the findings are in contrast with the results provided by Khani study (Khani et al., 2003) which was on the prevalence of hypertension in rural population of the city of Tarom. In this mentioned study, the rate of obesity among the population under study was higher than the obesity rate in the present study; however, in that study, those individuals who were obese had hypertension than those with a normal BMI. Considering high-fat food consumption, 27% of the case group and 35.8% of the controls were inclined to use high-fat foods. These results are somewhat similar to those found in the study by Moghimi (Moghimi et al., 2007), Obirikorang (Obirikorang et al., 2015) and Nkeh-Chungag (Nkeh et al., 2015)

The measures of PA, S, SM and TL are all significantly different, so that the obtained score in the dimensions listed among the patients with hypertension was significantly lower than the score obtained by the control group. The results are consistent with the results of the study by Wakasugi (2014). In our study, about half of the participants in both groups had announced that they had less than eight hours of sleepin one day; these results seem to be in harmony with reality due to the high average age of the participants. In Wakasugi’s study (Wakasugi’s, 2014), as well, the rate of sleep among the patients with hypertension had fallen.

In our study, 74% of participants had foods with moderate salt and 8.8% had salty foods. In the study by Lelong (Lelong et al., 2015); high salt intake was introduced as a risk factor in the development of hypertension, too.

The results of the present study showed that the mean scores of the case and control groups in terms of the dimensions of PA, S, SM and total lifestyle (TL) differed significantly, so that the total score in the mentioned dimensions among patients with hypertension was significantly lower than the score by healthy individuals. In a study by Najjar (Najar et al., 2005) the patients with hypertension had significant differences with the control group in terms of N, SM, sleep, smoking and BMI which was in consistency with our results. It is therefore necessary to have enough education and interventions for the patients with hypertension and other community members in order to adopt healthy lifestyles and behaviors and appropriate behavioral habits. These interventions should be initiated at a younger age because showing a behavior and becoming accustomed to it starts from childhood.

In our study, the mean total score and the aspects of PA, SM, N, IR and HR among the smokers was significantly lower than the score obtained by non-smokers. These results are in agreement with the studies by Wakasugi (Wakasugi’s et al., 2014), Saberi (Saberi et al., 2014), Gharipour (Gharipour et al., 2015) and Hu (Hu et al., 2015).

The logistic regression in the current study demonstrated that the relationship between lifestyle total score and lifestyle in the dimensions of PA, S and SM and hypertension is statistically significant. In a research study by Kemppaine (Kemppainen et al., 2011) in Japan and Behrouz (Behrouz et al., 2015), the participants in all aspects of N, HR, and IR had significant differences; but in the dimensions of N, SM and S, they had no significant differences. These results are not coordinated with the findings of the present study. This difference appears to be due to differences in the study population that is the study by Kemppainen (Kemppainen et al., 2011) was conducted in rural areas of northern Japan.

## 5. Conclusions

According to the results it seems that educational interventions in the field of healthy lifestyle for individuals with hypertension risk can have an effect on controlling this disease and reducing its incidence.
